# Taurine is a natural suppressor of urea cycle via targeting ASL

**DOI:** 10.1038/s41420-026-02959-6

**Published:** 2026-02-18

**Authors:** Keqiang Rao, Ke Zheng, Yunfan Sun, Jing He

**Affiliations:** 1 Department of Hepatobiliary Surgery and Liver Transplantation, Zhongshan Hospital, Fudan University, Shanghai, China; 2 Department of General Surgery, Zhongshan Hospital, Fudan University, Shanghai, China; 3 Department of Radiotherapy, The Affiliated Cancer Hospital of Zhengzhou University & Henan Cancer Hospital, Zhengzhou, China

**Keywords:** Cancer metabolism, Cancer metabolism

## Abstract

Hepatocellular carcinoma (HCC) has become the leading cause of global cancer-related mortality, which raises the demand for optimized therapeutic routes. The semi-essential micronutrient taurine has been gradually identified as a pivotal player linked to various diseases. Nevertheless, the metabolic impacts of taurine on hepatocellular carcinoma remain elusive. Here, we report that taurine is a negative regulator of urea cycle, thereby exerting a suppressive effect on growth of HCC tumors. Mechanistically, argininosuccinate lyase (ASL) is uncovered as the main target of taurine in repressing urea cycle of HCC cell lines. Furthermore, Fos proto-oncogene (FOS) functions as the transcription factor of ASL, which is significantly reduced upon taurine treatment. Physiologically, FOS-ASL axis is required for metabolic effects of taurine and contributes to growth of HCC tumors. Expression of ASL correlates with the inhibitory effect of taurine. Ultimately, synergistic blockade of glutaminolysis and urea cycle indicates that taurine is sufficient to substantially enhance the efficacy of the glutaminase GLS1 inhibitor in management of hepatocellular carcinoma. Collectively, these findings not only illustrate the metabolic mechanism of taurine in controlling growth of HCC tumors, but also create a promising route for utilization of taurine in clinic.

## Introduction

Liver cancer, mainly developed from smoking, virus infection, alcohol abuse, and so forth [1], contributes to the third leading-cause of cancer-related mortality [2]. Hepatocellular carcinoma, accounting for 75-85% of liver cancer, represents the major type of primary liver cancers [2]. Despite the fact that a plethora of pharmacological and surgical approaches have been utilized in management of early-stage HCC [3], more than 50% of determined HCC are diagnosed as advanced-stage HCC [4, 5], which highlights an urgent demand for more curative strategies to improve the status of HCC therapeutics.

Taurine, also known as 2‑aminoethanesulfonic acid, is a sulfur-containing amino acid and widely distributed across tissues [6, 7]. Taurine is also one of the most abundant free amino acids linked to multiple essential biological processes and health issues such as aging [8], diabetes [9], hypertension [10], liver diseases [11] and cancers [12]. In regard to cancers, taurine exerts a suppressive effect on proliferation of tumors. The BCL2 binding component PUMA has been uncovered as the critical target of taurine to initiate apoptosis of cancer cells [13–15]. The Hippo signaling core component MST1 is another effector of taurine to enhance apoptosis [16, 17]. Recently, the transporter SLC6A6 has been identified to license tumor cells to outcompete CD8^+^ T cells for taurine uptake, thus leading to exhaustion of CD8^+^ T cells and immune evasion [18]. Hence, taurine supplementation confers reinvigorated CD8^+^ T cells and enhances efficacy of therapy [18]. Nonetheless, taurine collaborated with proline to promote lung cancer development via enhancing Azgp1/mTOR signaling [19], implying the controversial role of taurine in various cancer types. Cancer cells always undergo concrete metabolic reprogramming to generate building blocks such as proteins, lipids and nucleotides, thus enabling robust proliferation [20]. Nevertheless, whether taurine plays a pivotal role in orchestrating the development of HCC is uncertain.

Nitrogen disposal is vital for cancer cells to achieve rapid growth and proliferation. Urea cycle, primarily taking place in the liver, is the major route to convert toxic ammonia originated by glutaminolysis into secreted urea [21]. Besides, urea cycle is the sole source of endogenous arginine [22], which provides substrates and precursors for biosynthesis of polyamines and proteins. Considering the essentiality of arginine to cancer development and progression, various arginine-deprivation therapies (ADT) such as pegylated arginine deiminase (ADI-PEG20) and PEGylated recombinant human ARG1 (PEG-rhARG1) have been explored for the therapy of cancers [23, 24]. The argininosuccinate lyase ASL is the unique enzyme responsible for cleavage of argininosuccinate to generate arginine [25]. In addition, the byproduct fumarate catalyzed by ASL, fuels TCA cycle to meet the demand of rapid proliferation [26].

Here we reported that taurine constrains urea cycle via repressing transcription of *ASL* in HCC cell lines. FOS is identified as the transcription factor of *ASL*. Furthermore, deletion of FOS disrupts taurine-derived suppression of *ASL* expression. In regard to metabolic aspects, taurine rewires the production of urea cycle metabolites. Physiologically, ASL plays a vital role in mediating taurine-induced arrest of HCC tumor growth. Synergistic blockade of glutaminolysis and urea cycle substantially impairs growth of HCC tumors. These findings not only put forth a heretofore unrecognized suppressive mechanism of urea cycle originated by taurine, but also propose implications for HCC therapy in clinic.

## Results

### Taurine negatively regulates urea cycle

**Fig. 1:**
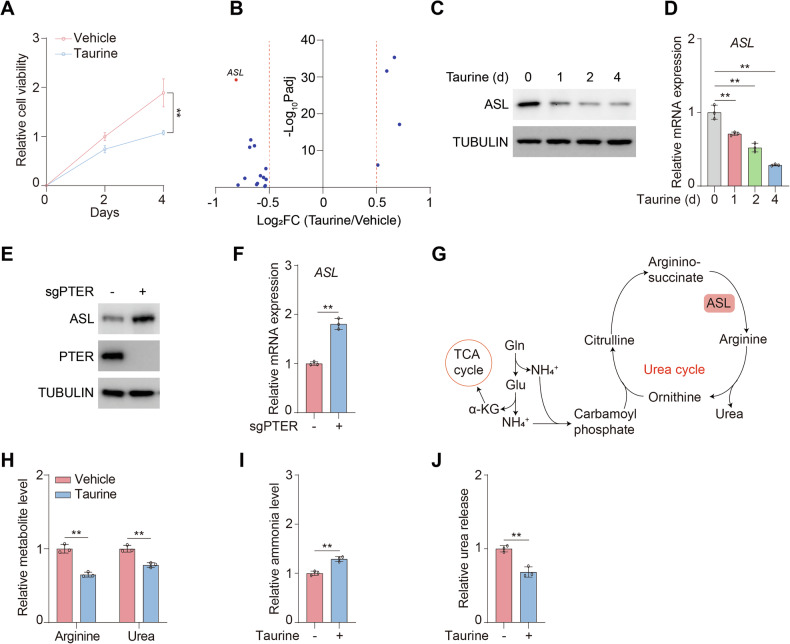
Taurine negatively regulates urea cycle. **A** Cell viability of MHCC97H cells treated with or without 100 μM taurine for the indicated days were analyzed. **B** RNAseq demonstrated that the expression of *ASL* was significantly changed among various metabolic enzymes in MHCC97H cells treated with or without 100 μM taurine for four days. Volcano plot was displayed with log_2_FC > 0.5 or <-0.5. **C**, **D** MHCC97H cells were treated with or without 100 μM taurine for the indicated days. Immunoblotting analysis was performed using the indicated antibodies (**C**). mRNA levels of *ASL* were analyzed (**D**). **E**, **F** MHCC97H cells were transfected with or without sgRNA targeting PTER (sgPTER). Immunoblotting analysis was performed using the indicated antibodies (**E**). mRNA levels of *ASL* were analyzed (**F**). **G** Schematic of ASL-mediated urea cycle. **H**-**J** MHCC97H cells were treated with or without 100 μM taurine for four days. Arginine and urea levels (**H**) ammonia levels (**I**) and urea release (**J**) were analyzed. Data are presented as mean ± SD, *n* = 3 independent repeats. Unpaired, two-tailed *t* test; ^**^P < 0.01.

Since the abundance of taurine in complete media (Fig. S1A) was far below the physiological concentration [8], to explore the role of taurine on cell proliferation, we treated hepatocellular carcinoma cancer cells MHCC97H and HepG2 with 100 μM taurine for consecutive days and found that taurine was sufficient to restrain cell proliferation (Figs. 1A and S1B). To reveal the molecular mechanism in depth, we conducted RNAseq analysis and found that *ASL*, the key enzyme of urea cycle, was strikingly decreased upon taurine treatment (Fig. 1B). To validate the results, we found that the protein (Figs. 1C and S1C) and mRNA (Figs. 1D and S1D) levels of ASL was gradually declined under taurine treatment.

PTER has been demonstrated as N-acetyltaurine hydrolase to generate taurine and regulate obesity [27]. We therefore established PTER knockout (sgPTER) HCC cell lines (Figs. 1E and S1E) and found that loss of PTER enhanced expression of ASL (Figs. 1E, F and S1E, F). Since ASL is required for maintaining homeostasis of urea cycle, which is the major route for ammonia disposal and production of arginine and urea (Fig. 1G), we examined the cellular metabolites and found that taurine largely reduced cellular arginine and urea levels (Figs. 1H and S1G). Consistently, taurine prominently enhanced ammonia accumulation (Figs. 1I and S1H). As a result, urea released was disrupted by taurine (Figs. 1J and S1I). Taken together, taurine represses urea cycle and expression of ASL.

### Metabolic impacts of taurine are linked to expression of ASL

**Fig. 2:**
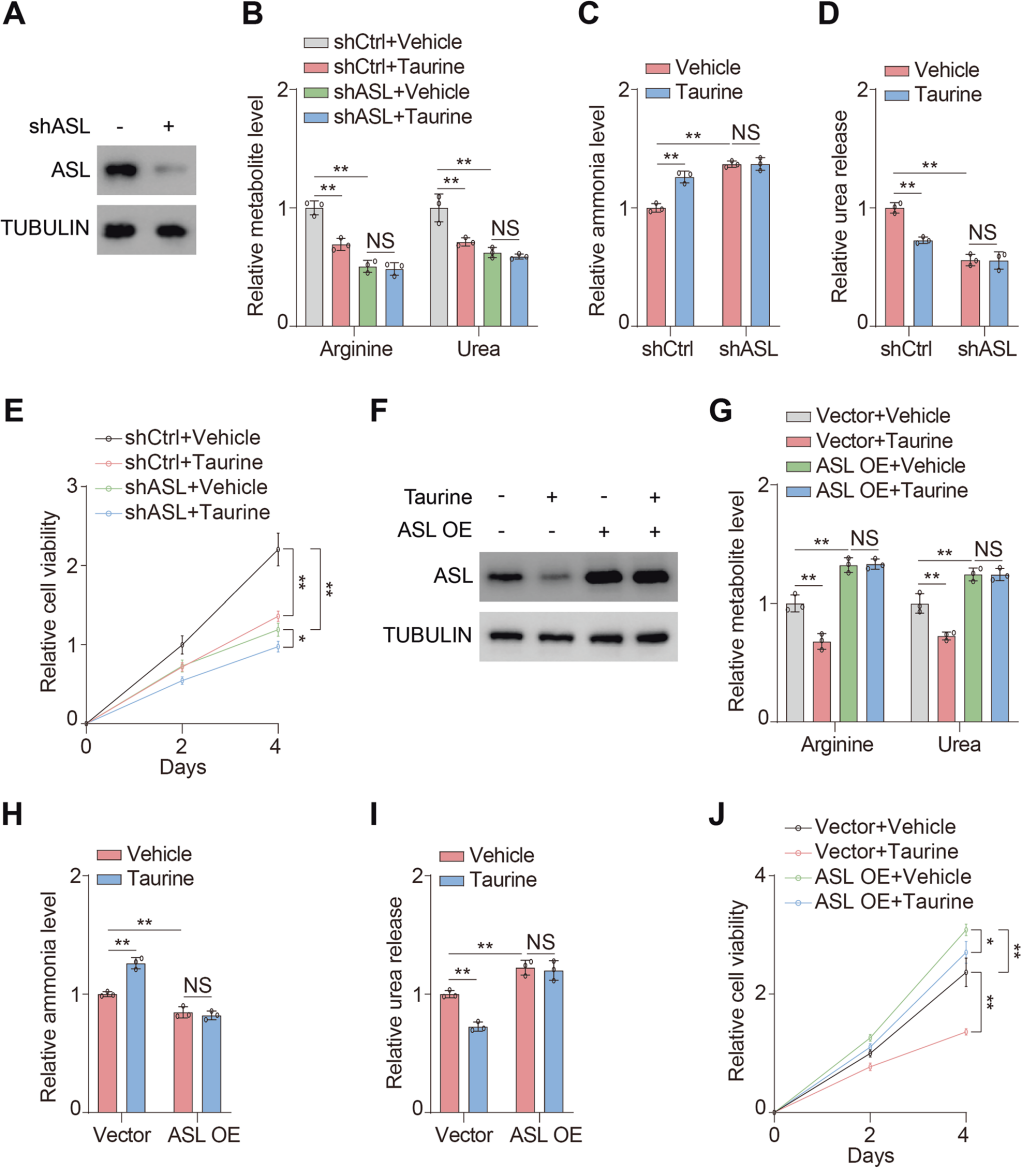
Metabolic impacts of taurine are linked to expression of ASL. **A** MHCC97H cells were transfected with or without shRNA targeting ASL (shASL). Immunoblotting analysis was performed using the indicated antibodies. **B**-**D** shCtrl and shASL MHCC97H cells were treated with or without 100 μM taurine for four days. Arginine and urea levels (**B**) ammonia levels (**C**) and urea release (**D**) were analyzed. **E** Cell viability of shCtrl and shASL MHCC97H cells treated with or without 100 μM taurine for indicated days were analyzed. **F** MHCC97H cells were stably overexpressed with or without ASL (ASL OE) and treated with or without 100 μM taurine for two days. Immunoblotting analysis was performed using the indicated antibodies. **G**-**I** Control and ASL OE MHCC97H cells were treated with or without 100 μM taurine for two days. Arginine and urea levels (**G**) ammonia levels (**H**) and urea release (**I**) were analyzed. **J** Cell viability of control and ASL OE MHCC97H cells treated with or without 100 μM taurine for indicated days were analyzed. Data are presented as mean ± SD, *n* = 3 independent repeats. Unpaired, two-tailed *t* test; ^*^P < 0.05; ^**^P < 0.01. NS, not significant.

To verify whether ASL mediated metabolic impacts of taurine, we conducted ASL knockdown (shASL) HCC cell lines (Figs. 2A and S2A). The results showed that knockdown of ASL compromised taurine-induced reduction of arginine and urea levels (Figs. 2B and S2B). Likewise, the ammonia levels (Figs. 2C and S2C) and urea release (Figs. 2D and S2D) were accordingly orchestrated by taurine in an ASL-dependent manner. Consequently, ASL was involved in taurine-mediated inhibition of cell proliferation. Compared with control group, loss of ASL presented mild reduction of cell viability upon taurine treatment (Figs. 2E and S2E).

Next, we generated HCC cell lines stably expressing ASL, which were identified resistant to taurine-induced suppression during transient treatment (Figs. 2F and S2F). Overexpression of ASL significantly facilitated arginine and urea production in control group (Figs. 2G and S2G). Moreover, ASL was capable of sustaining urea cycle in response to taurine (Figs. 2G and S2G). Besides, overexpression of ASL maintained ammonia consumption regardless of taurine (Figs. 2H and S2H). Accordingly, urea release was enhanced by ASL, whereas taurine failed to impair urea release under forced expression of ASL (Figs. 2I and S2I). In regard to cell viability, overexpression of ASL accelerated cell proliferation and resisted the suppressive effect of taurine compared with the control group (Figs. 2J and S2J). ASL functioned as the critical enzyme in urea cycle to catalyze arginine and urea generation (Fig. 1G) and enabled tumor growth [22, 28]. Moreover, a previous study has demonstrated that sole knockdown of ASL is sufficient to disrupt urea cycle and proliferation of HCC cells [22]. These findings indicated that manipulation of ASL expression enables the orchestration of urea cycle. Considering that ASL has been revealed as a crucial target of taurine and was responsible for the suppressive effect on urea cycle caused by taurine, exogenous forced expression of ASL was capable of maintaining urea cycle homeostasis and cell viability and presented resistant to taurine treatment. Collectively, ASL plays a pivotal role in mediating the metabolic impacts of taurine.

### Taurine represses expression of ASL via FOS

**Fig. 3:**
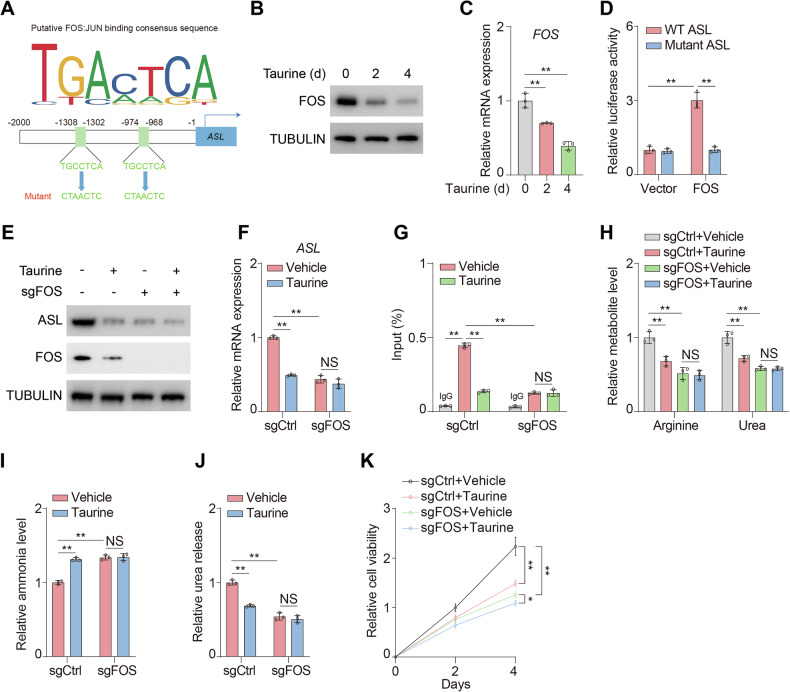
Taurine represses expression of ASL via FOS. **A** Putative FOS:JUN binding consensus sequences in promoter of *ASL* (bottom) were compared with motif predicted using JASPAR database (top). **B**, **C** MHCC97H cells were treated with or without 100 μM taurine for the indicated days. Immunoblotting analysis was performed using the indicated antibodies (**B**). mRNA levels of *FOS* were analyzed (**C**). **D** Plasmid containing either WT or mutant promoter sequence of *ASL* was co-transfected with FOS into 293T cells. Dual luciferase reporter assay was performed. **E**, **F** MHCC97H cells were transfected with or without sgRNA targeting FOS (sgFOS) and treated with or without 100 μM taurine for four days. Immunoblotting analysis was performed using the indicated antibodies (**E**). mRNA levels of *ASL* were analyzed (**F**). **G** ChIP assay was performed in sgCtrl and sgFOS MHCC97H cells using antibodies against c-JUN. DNA enrichment was examined by quantitative real-time PCR. The *y* axis shows the value normalized to input. **H**-**J** sgCtrl and sgFOS MHCC97H cells were treated with or without 100 μM taurine for four days. Arginine and urea levels (**H**) ammonia levels (**I**) and urea release (**J**) were analyzed. **K** Cell viability of sgCtrl and sgFOS MHCC97H cells treated with or without 100 μM taurine for indicated days were analyzed. Data are presented as mean ± SD, *n* = 3 independent repeats. Unpaired, two-tailed *t* test; ^*^P < 0.05; ^**^P < 0.01. NS, not significant.

To decipher the mechanism of taurine-triggered suppression of *ASL* transcription, we predicted the putative transcription factor of *ASL* and found that ASL promoter harbored two consensus binding sites of FOS:JUN complex (Fig. 3A). Interestingly, RNAseq revealed that taurine remarkably restrained expression of *FOS*, which provoked us to explore whether FOS:JUN complex could initiate transcription of *ASL*. In line with the RNAseq results, the expression of FOS was substantially reduced by taurine (Figs. 3B, C and S3A, B). Furthermore, luciferase reported assay assured FOS could associate with *ASL* promoter, while mutation of the putative sites entirely blocked the interaction (Fig. 3D).

Next, FOS knockout (sgFOS) HCC cell lines were carried out (Figs. 3E and S3C). As a result, depletion of FOS impaired expression of ASL, while compare with control group (sgCtrl), taurine exerted minor effect on expression of ASL in sgFOS group (Figs. 3E, F and S3C, D). Moreover, chromatin immunoprecipitation (ChIP) analysis indicated that c-JUN was abundantly enriched on the promoter of *ASL*, which was compromised upon loss of FOS (Figs. 3G and S3E). In regard to the metabolic effects, deficiency of FOS notably impaired arginine and urea production, which phenocopied the effect of taurine treatment (Figs. 3H and S3F). Moreover, loss of FOS disrupted the reduction of arginine and urea caused by taurine (Figs. 3H and S3F). Consistently, taurine coordinated ammonia abundance (Figs. 3I and S3G) and urea release (Figs. 3J and S3H) in a FOS-dependent manner. Consequently, deletion of FOS significantly suppressed cell viability, while taurine exerted a mildly suppressive effect on proliferation capacity of sgFOS cells (Figs. 3K and S3I). Together, FOS is critical for taurine-mediated suppression of urea cycle.

### FOS-ASL axis contributes to metabolic effects of taurine

**Fig. 4:**
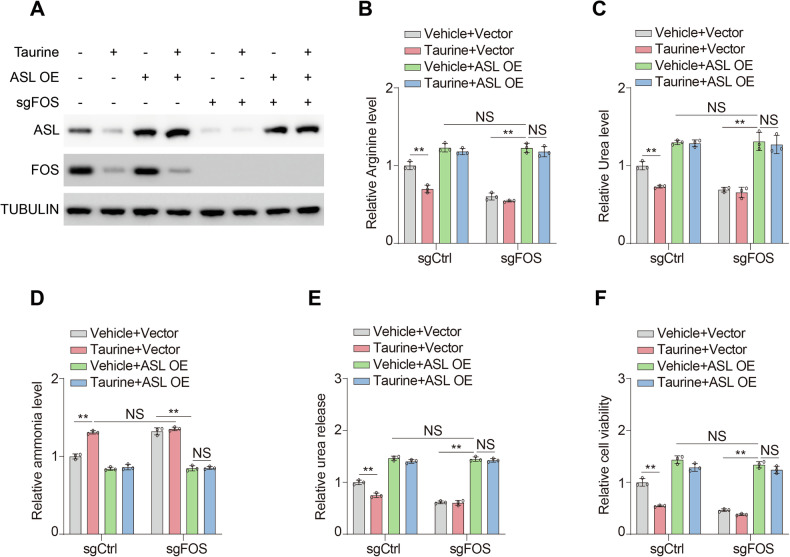
FOS-ASL axis contributes to metabolic effects of taurine. **A**-**F** MHCC97H cells were transfected with or without sgRNA targeting FOS (sgFOS), overexpressed with or without ASL (ASL OE) and treated with or without 100 μM taurine for two days. Immunoblotting analysis was performed using the indicated antibodies (**A**). Arginine (**B**), urea (**C**), ammonia levels (**D**), urea release (**E**), and cell viability (**F**) were analyzed. Data are presented as mean ± SD, *n* = 3 independent repeats. Unpaired, two-tailed *t* test; ^**^P < 0.01. NS, not significant.

To validate the role of FOS-ASL axis in mediating the metabolic impacts of taurine, we tried to introduce ectopic ASL into FOS knockout cells (Figs. 4A and S4A). Overexpression of ASL considerably rescued arginine (Figs. 4B and S4B) and urea (Figs. 4C and S4C) production under either taurine treatment or deletion of FOS. Moreover, ASL could entirely support urea cycle in FOS knockout cells with taurine treatment (Figs. 4B, C and S4B, C), implying the FOS-ASL axis was vital for taurine in regulation of urea cycle. Likewise, forced expression of ASL reversed the ammonia accumulation (Figs. 4D and S4D) and urea release (Figs. 4E and S4E) caused by taurine or FOS knockout. Ultimately, overexpression of ASL fueled cell proliferation regardless of taurine treatment or loss of FOS (Figs. 4F and S4F). These findings indicated that FOS-ASL axis contributes to metabolic effects of taurine.

### Taurine enhances the efficacy of glutaminolysis blockade

**Fig. 5:**
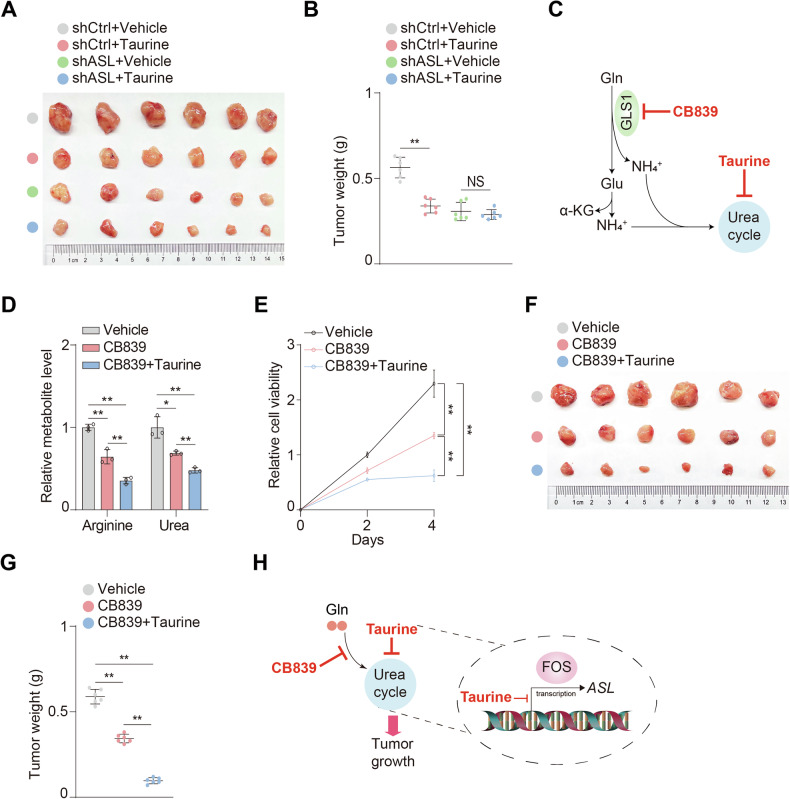
Taurine enhances the efficacy of glutaminolysis blockade. **A**, **B** shCtrl and shASL MHCC97H cells were subcutaneously injected into athymic nude mice administrated with or without taurine. Representative tumor xenografts (**A**). Mice were sacrificed in week 4 and weight were calculated (**B**). *n* = 6 independent animals. **C** Schematic of GLS1-mediated glutaminolysis, which can be blocked by its inhibitor CB839. **D** MHCC97H cells were treated with or without 500 nM CB839 or 100 μM taurine for four days. Arginine and urea levels were analyzed. **E** Cell viability of MHCC97H cells treated with or without 500 nM CB839 or 100 μM taurine for indicated days were analyzed. **F**, **G** MHCC97H cells were subcutaneously injected into athymic nude mice administrated with or without CB839 or taurine. Representative tumor xenografts (**F**). Mice were sacrificed in week 4 and weight were calculated (**G**). *n* = 6 independent animals. **H** Schematic of synergistic blockade of glutaminolysis and urea cycle to suppress tumor growth. Data are presented as mean ± SD, *n* = 3 independent repeats. Unpaired, two-tailed *t* test; ^*^P < 0.05; ^**^P < 0.01. NS, not significant.

Subsequently, the physiological consequences of taurine-mediated metabolic impacts were explored via xenograft analysis. The results showed that taurine solely was sufficient to suppress tumor growth (Fig. 5A, B). Compared with control group, taurine failed to exert an additional suppressive effect on tumors with knockdown of ASL (Fig. 5A, B), suggesting ASL was the main metabolic target in orchestrating tumor growth.

Since glutaminolysis catalyzed by GLS1 fuels TCA cycle, urea cycle and other physiological provesses via producing α-KG and ammonia (Fig. 5C), inhibition of glutaminolysis has been considered as a promising route for cancer therapy [29]. Nevertheless, due to the limited efficacy of GLS1 inhibitor, there is an urgent demand to improve the efficacy. Recently, preclinical studies have revealed that radiation therapy and drugs targeting redox stress have been utilized to synergize with GLS1 inhibitor for cancer therapy [30, 31]. Since glutamine is the major source of ammonia to fuel urea cycle [32], we proposed that dual blockade of glutaminolysis and urea cycle may be a promising route for cancer therapy. CB839 (Fig. 5C), the inhibitor of GLS1 which catalyzes the first step of glutaminolysis [33], was employed to verify the hypothesis. Firstly, we found that either taurine or CB839 exerted no effect on GLS1 expression (Fig. S5A, B) and its endogenous activity as indicated by cellular glutamate/glutamine ratio (Glu/Gln) (Fig. S5C) or ASL expression (Fig. S5D, E), respectively. Interestingly, CB839 could notably dampen urea cycle capacity, which was strengthened by taurine (Fig. 5D). In line with this, CB839 plus taurine substantially impaired cell viability compared with CB839 solely (Fig. 5E). In regard to in vivo validation using xenograft model, compared with CB839 group, synergistic combination of CB839 and taurine exhibited a prominent inhibitory effect on tumor growth (Fig. 5F, G). Together, these results demonstrated that taurine could enhance the efficacy of glutaminolysis blockade to repress tumor growth (Fig. 5H).

## Discussion

In the present study, we reported that the amino acid taurine is sufficient to suppress urea cycle and expression of its key enzyme ASL. Moreover, we found that FOS functions as the transcription factor of *ASL*. The expression of FOS is substantially decreased upon taurine treatment. FOS-ASL axis contributes to the metabolic impacts of taurine. Loss of ASL or FOS largely compromises the tumor suppressive role of taurine. Finally, synergistic blockade of glutaminolysis and urea cycle using CB839 and taurine could dramatically restrict tumor growth. These findings not only illustrate the molecular mechanism of the regulatory role of taurine in cell metabolism but also provide a promising strategy for cancer therapy.

Taurine has been gradually revealed as an essential amino acid to participate in various physiological processes such as aging [8], obesity [27] and tumorigenesis [18]. Nevertheless, the metabolic effect of taurine remains unidentified, noting that metabolic dysregulation has become an emerging hallmark of cancers [34]. Our study indicated that urea cycle is a vital target of taurine, mainly via inhibiting FOS-mediated transcription of *ASL*. Since metabolites have been demonstrated to regulate gene expression through various routes, such as transcriptional, post-transcriptional, translational, or post-translational levels, the concrete mechanism of taurine-induced suppression of FOS expression remains to be further explored. Lately, taurine from tumor microenvironment was uncovered to drive glycolysis and enable leukaemogenesis [35], suggesting the physiological role of taurine is context-dependent and shaped by tumor types and local niches.

Recently, taurine has been utilized to alleviate aging [36] and chronic liver disease [37] in clinical trial, which highlights the application possibility of taurine in clinic. Considering taurine is a common amino acid and easily obtained, especially through energetic beverages, taurine might be an ideal and safe adjuvant to enhance the efficacy of cancer therapies that target glutaminolysis or urea cycle. Our findings have proposed that taurine is a promising adjuvant to enhance the efficacy of therapies targeting glutaminolysis, which may benefit potential clinical strategy development.

## Methods

### Cell culture

MHCC97H, HepG2 and 293T cells were maintained in DMEM (Gibco, 11965092) supplemented with 10% FBS (Biological Industries, C04001). All cell lines were authenticated by short tandem repeat fingerprinting and routinely tested for mycoplasma contamination. For taurine treatment, 100 μM taurine was added to DMEM media with 10% FBS.

### Antibodies

Antibodies that recognize TUBULIN (11224-1-AP) and ASL (16645-1-AP) were purchased from Proteintech. Antibodies that recognize GLS1 (56750), FOS (2250S) and c-Jun (9165S) were purchased from Cell Signaling Technology. Antibody that recognizes PTER (PA5-20750) was purchased from Thermo Fisher.

### Materials

Taurine (T0022) and protease inhibitor cocktail (C0001) were purchased from TargetMol. Cellular ammonia (A086-1-1), urea release (C013-2-1) and glutamate (A074-1-1) assay kit were purchased from NJJCBIO. Glutamine assay kit (YS111081) was purchased from Y-J Biological. CB839 (HY-12248) was purchased from MedChemExpress. EZ Protein any KD PAGE kit (AP15L535) was purchased from Life-iLab. ChIP assay kit (56383) was purchased from Cell Signaling Technology. Dual Luciferase Reporter Gene Assay Kit (RG027) was purchased from Beyotime. RNA purification (B0004D) and reverse transcription (EZB-RT2G) kit were purchased from EZBioscience. SYBR qPCR mix (11201ES) was purchased from Yeasen. CCK8 assay kit (C6005) was purchased from New Cell & Molecular Biotech. Taurine assay kit (MET-5071) was purchased from Cell Biolabs.

### DNA construction and transfection

The sgRNAs were generated by annealed oligonucleotides and cloned into pLentiCRISPRv2. The target sequence of *PTER* was GATGGAACCAGTATCAAGTG-3. The target sequence of *FOS* was GGGCTTCAACGCAGACTACG-3. The shRNA was generated by annealed oligonucleotides and cloned into pLKO.1. The target sequence of *ASL* was CCCATCATGGAGAAGTTCA-3. Plasmids were transfected into cells with EZ Trans (Life-iLab, AC04L092) following the instructions.

### Cell viability assay

1 × 10^3^ cells per well were seeded in 96-well plates for assay. Next, cells were incubated with 10% CCK-8 reagent that was diluted in DMEM at 37 °C for 1 h. Absorbance at 450 nm was acquired on indicated days using a microplate reader. Cell viability was calculated as normalized to the control group.

### Ammonia and urea release measurement

Cellular ammonia was measured following the instructions of quantification kit after cell lysate collection. Urea release was measured following the instructions of quantification kit after media collection.

### Glutamine and glutamate measurement

Glutamine and glutamate were measured following the instructions of quantification kits after cell lysate collection. Glutamine/glutamate ratio was calculated to indicate GLS1 activity.

### FOS:JUN motif analysis

Two-kilobase-long DNA sequences upstream of the transcriptional start site of *ASL* were retrieved using the UCSC Genome Browser (http://genome.ucsc.edu/). The DNA sequence was then aligned to consensus DNA binding sequence of FOS:JUN through the JASPAR database (https://jaspar.genereg.net/).

### ChIP assay

The ChIP assay was performed according to the manufacturer’s instructions. Briefly, 5 × 10^7^ cells per group were collected and fixed with formaldehyde. After that, chromatin was fragmented by sonication. Antibodies against c-Jun (1:50 dilution) were used for IP. qPCR was used to measure the amount of bound DNA, and the value of enrichment was calculated normalized to IgG. Primers covering the c-JUN binding site of *ASL* gene promoter region were used as followings: 5′-CCTCCACCTCCCTGGTTCAAGTGATTCTCC-3′ (forward) and 5′-GGTGCCTGCAATCCCAGCGACTCCGGAGGC-3′ (reverse).

### Gene expression analysis

Total RNA was isolated according to the manufacturer’s instructions. 1 μg of total RNA was used for cDNA generation. The cDNA in triplicate was assessed for target mRNA levels by qPCR with SYBR qPCR Master Mix (Yeasen, 11201ES08). We calculated relative mRNA levels normalized to human *TUBULIN* levels in the same samples. The qPCR primer sequences were: *ASL*: 5′- GCCGAGATGGACCAGATACTC-3′ (forward) and 5′- CTGCCGTTGCACCAATGAG-3′ (reverse); *FOS*: 5′- CCGGGGATAGCCTCTCTTACT-3′ (forward) and 5′- CCAGGTCCGTGCAGAAGTC-3′ (reverse); *TUBULIN*: 5′- TGGACTCTGTTCGCTCAGGT-3′ (forward) and 5′- TGCCTCCTTCCGTACCACAT-3′ (reverse).

### Luciferase reporter assay

The *ASL* promoter sequence (−2000 bp to 0 bp) was inserted into the pGL3 vector. The putative FOS:JUN binding consensus sequence involved in promoter was mutated into CTAACTC. Plasmid containing either WT or mutant promoter sequence was co-transfected with FOS into 293T cells. The luciferase activity was measured using a Dual Luciferase Reporter Gene Assay Kit.

### Animal study

MHCC97H cells were washed twice with PBS and concentrated to 10^6^ per 100 μl in PBS. Then, 100 μl of cells was subcutaneously injected into right back flank of 6-week-old male BALB/c nude mice. CB839 (200 mg/kg orally every 2 days) and taurine (100 mg/kg orally every 2 days) treatment was performed at day 7 after transplantation. Tumor size must not exceed 20 mm at the largest diameter in an adult mouse, and no experiments in this study generated a tumor burden over this limit. Mice were maintained in a temperature-controlled and light-controlled environment with ad libitum access to water. The mice were randomly put into separate groups for experiments and received a standard chow diet. All animal experiments were conducted under the guidelines and approved by the Institutional Animal Care and Use Committee of Zhongshan Hospital, Fudan University.

### Statistics and reproducibility

Statistical testing was performed using the unpaired, two-tailed Student’s *t*-test. All experiments were performed at least three times unless otherwise indicated, and representative results are shown in the figures. Figure legends indicate the N numbers. Analyzes were performed with GraphPad Prism. Data are presented as mean ± SD *P* values < 0.05 were considered statistically significant (^*^P < 0.05, ^**^P < 0.01). NS represents not significant.

## Supplementary information

Supplementary figure legends

Uncropped western blots

## Data Availability

RNA sequencing datasets are available at the NCBI SRA database under accession number PRJNA1244449.
