# Supplementary material for: Taurine is a natural suppressor of urea cycle via targeting ASL
Source: Cell Death Discov. 2026 Feb 18;12:99. doi: 10.1038/s41420-026-02959-6 (PMC12921272; doi:10.1038/s41420-026-02959-6)
Supplement: Supplementary file 1 — Supplementary figure legends [file 41420_2026_2959_MOESM1_ESM.docx]

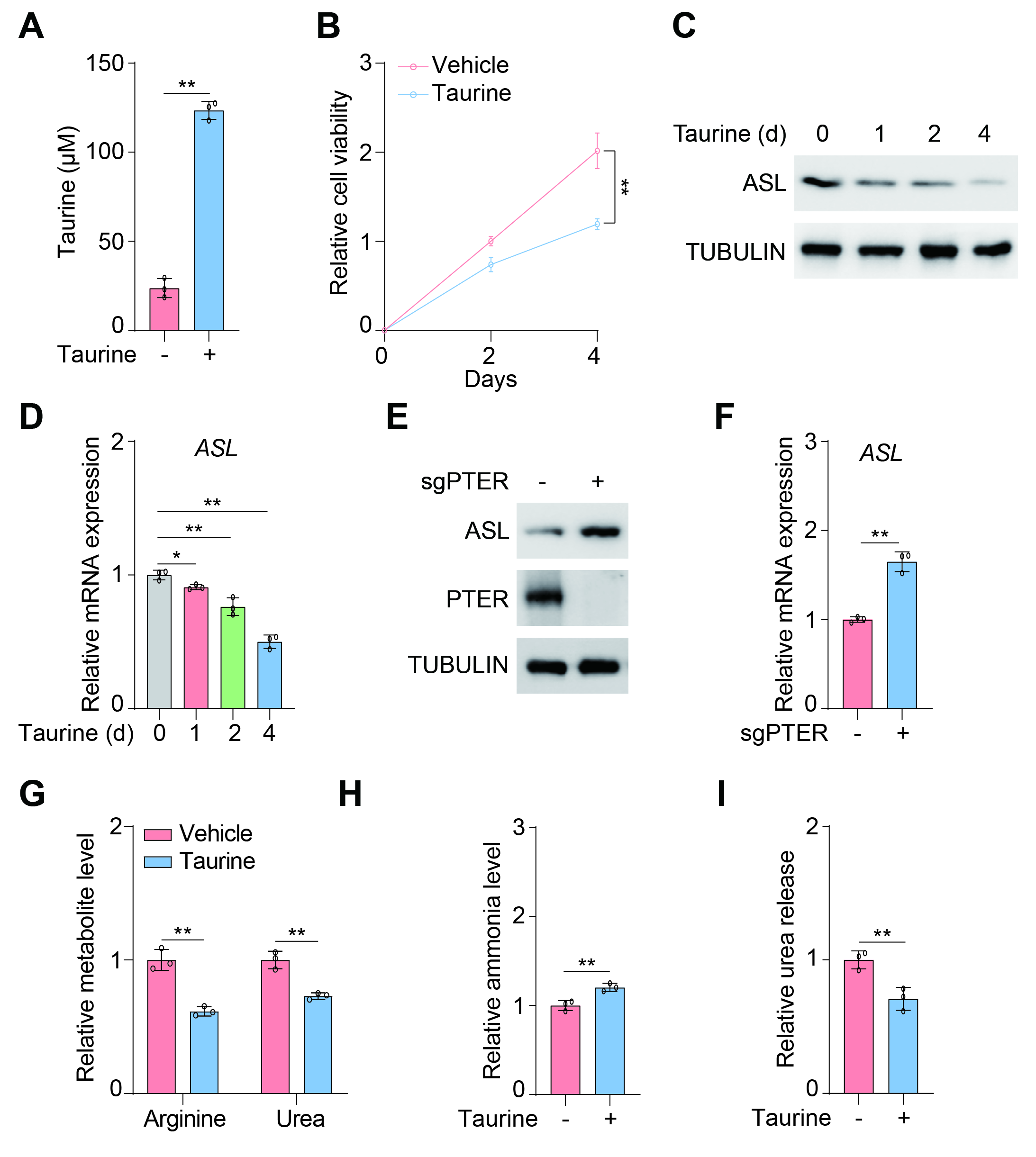


Fig. S1 Taurine is a negative regulator of urea cycle. (A) Taurine concentration was measured in DMEM containing 10% FBS treated with or without 100 μM taurine. (B) Cell viability of HepG2 cells treated with or without 100 μM taurine for the indicated days were analyzed. (C, D) HepG2 cells were treated with or without 100 μM taurine for the indicated days. Immunoblotting analysis was performed using the indicated antibodies (C). mRNA levels of *ASL* were analyzed (D). (E, F) HepG2 cells were transfected with or without sgRNA targeting PTER (sgPTER). Immunoblotting analysis was performed using the indicated antibodies (E). mRNA levels of *ASL* were analyzed (F). (G, H, I) HepG2 cells were treated with or without 100 μM taurine for four days. Arginine and urea levels (G), ammonia levels (H) and urea release (I) were analyzed. Data are presented as mean ± SD, n = 3 independent repeats. Unpaired, two-tailed t test; **P* < 0.05; ***P* < 0.01.


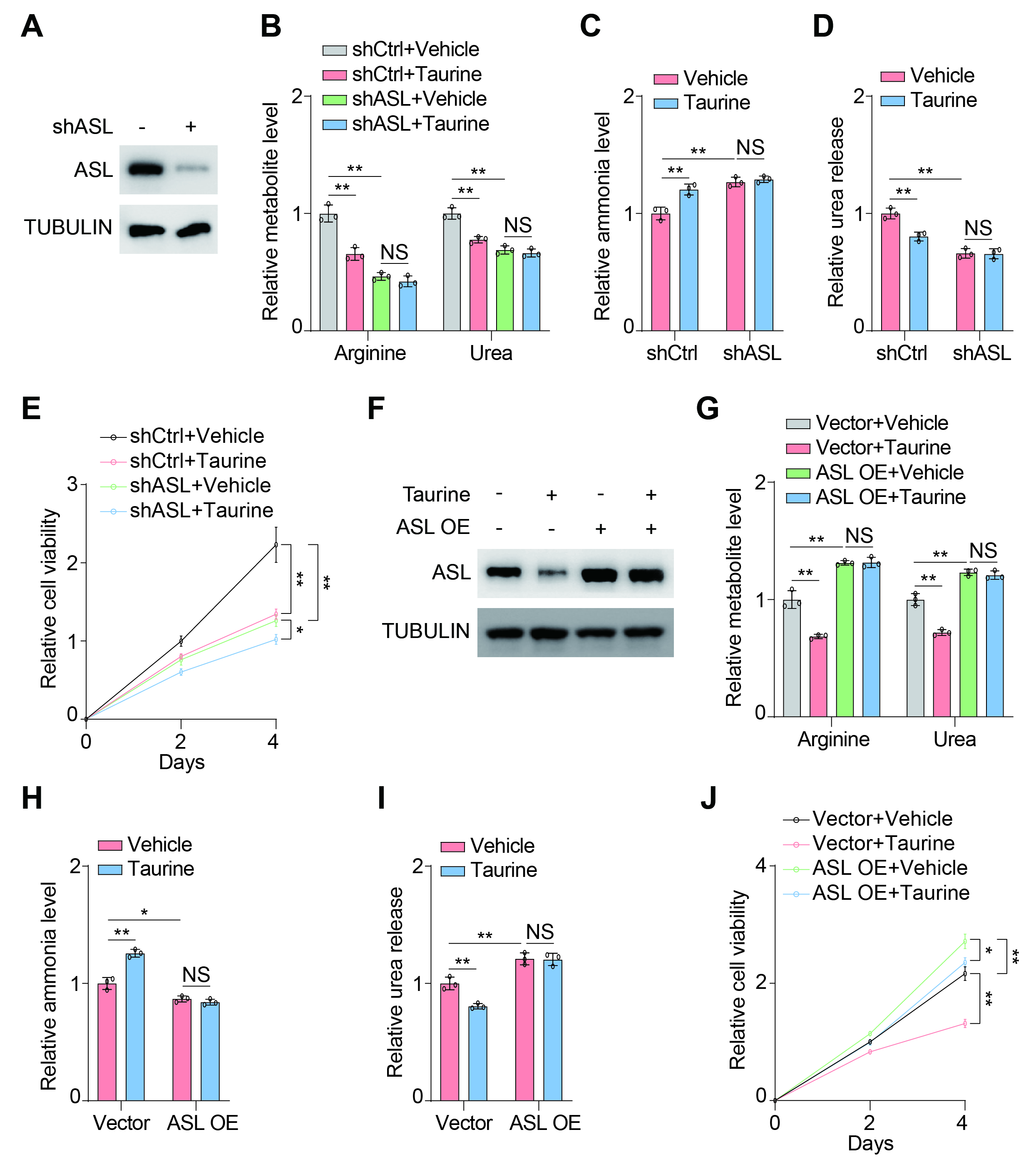


Fig. S2 ASL is vital for the metabolic impacts of taurine. (A) HepG2 cells were transfected with or without shRNA targeting ASL (shASL). Immunoblotting analysis was performed using the indicated antibodies. (B, C, D) shCtrl and shASL HepG2 cells were treated with or without 100 μM taurine for four days. Arginine and urea levels (B), ammonia levels (C) and urea release (D) were analyzed. (E) Cell viability of shCtrl and shASL HepG2 cells treated with or without 100 μM taurine for indicated days were analyzed. (F) HepG2 cells were stably overexpressed with or without ASL (ASL OE) and treated with or without 100 μM taurine for two days. Immunoblotting analysis was performed using the indicated antibodies. (G, H, I) Control and ASL OE HepG2 cells were treated with or without 100 μM taurine for two days. Arginine and urea levels (G), ammonia levels (H) and urea release (I) were analyzed. (J) Cell viability of control and ASL OE HepG2 cells treated with or without 100 μM taurine for indicated days were analyzed. Data are presented as mean ± SD, n = 3 independent repeats. Unpaired, two-tailed t test; **P* < 0.05; ***P* < 0.01. NS, not significant.


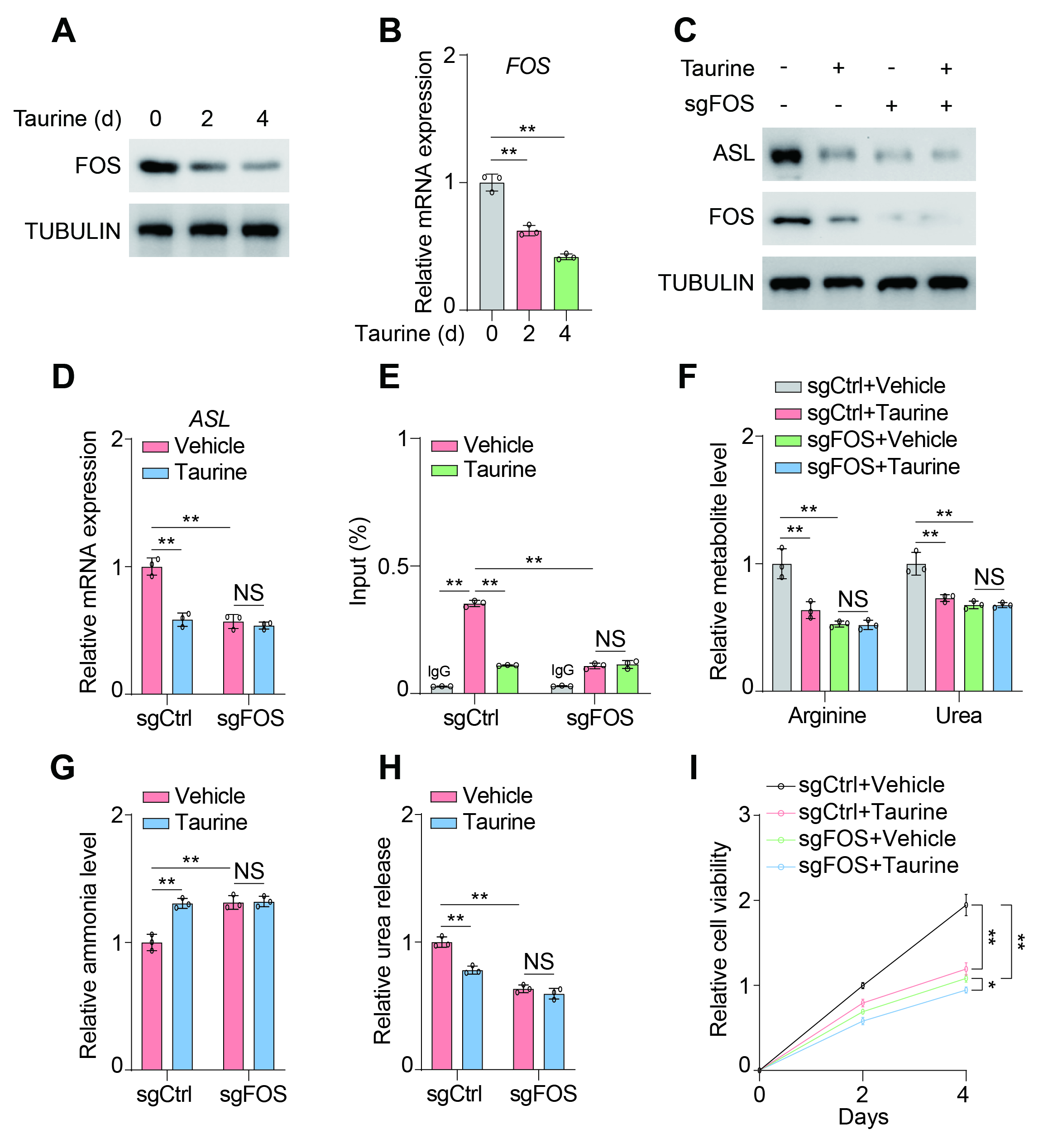


Fig. S3 FOS mediates ASL expression under taurine treatment. (A, B) HepG2 cells were treated with or without 100 μM taurine for the indicated days. Immunoblotting analysis was performed using the indicated antibodies (A). mRNA levels of *FOS* were analyzed (B). (C, D) HepG2 cells were transfected with or without sgRNA targeting FOS (sgFOS) and treated with or without 100 μM taurine for four days. Immunoblotting analysis was performed using the indicated antibodies (C). mRNA levels of *ASL* were analyzed (D). (E) ChIP assay was performed in sgCtrl and sgFOS HepG2 cells using antibodies against c-JUN. DNA enrichment was examined by quantitative real-time PCR. The y axis shows the value normalized to input. (F, G, H) sgCtrl and sgFOS HepG2 cells were treated with or without 100 μM taurine for four days. Arginine and urea levels (F), ammonia levels (G) and urea release (H) were analyzed. (I) Cell viability of sgCtrl and sgFOS HepG2 cells treated with or without 100 μM taurine for indicated days were analyzed. Data are presented as mean ± SD, n = 3 independent repeats. Unpaired, two-tailed t test; **P* < 0.05; ***P* < 0.01. NS, not significant.


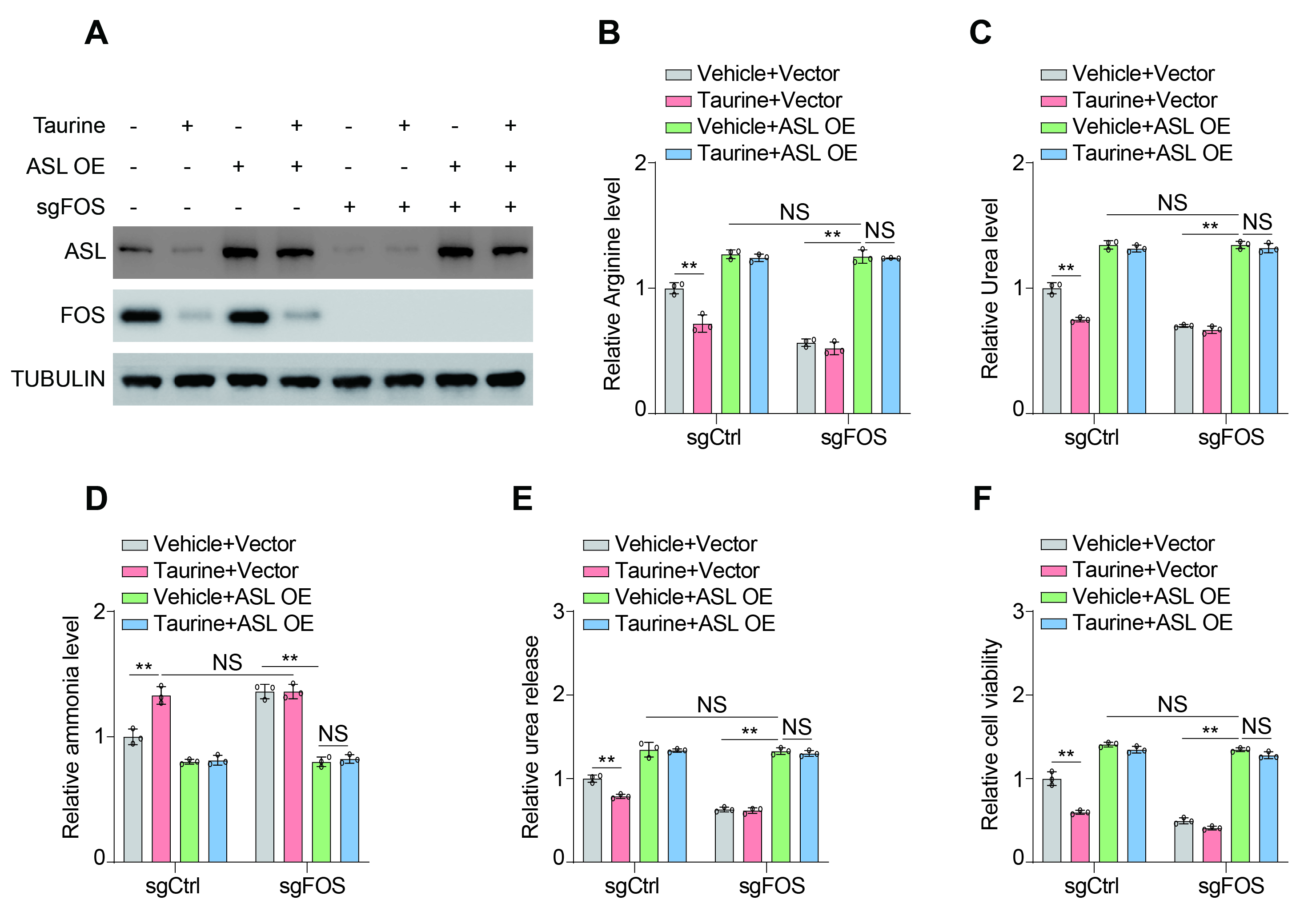
Fig. S4 FOS-ASL axis is essential for the metabolic effects of taurine. (A, B, C, D, E, F) HepG2 cells were transfected with or without sgRNA targeting FOS (sgFOS), overexpressed with or without ASL (ASL OE) and treated with or without 100 μM taurine for two days. Immunoblotting analysis was performed using the indicated antibodies (A). Arginine (B), urea (C), ammonia levels (D), urea release (E) and cell viability (F) were analyzed. Data are presented as mean ± SD, n = 3 independent repeats. Unpaired, two-tailed t test; ***P* < 0.01. NS, not significant.


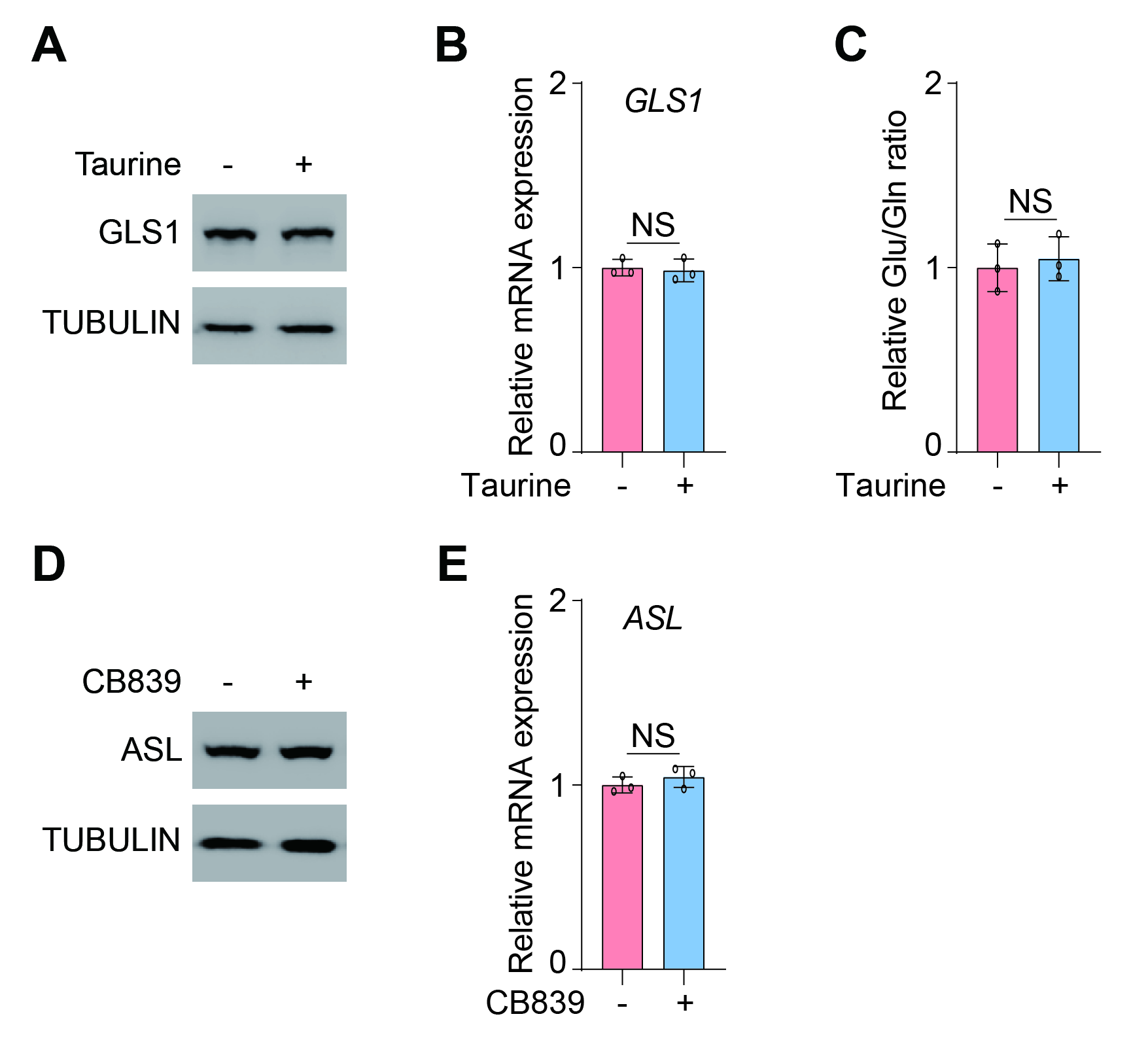


Fig. S5 Taurine negatively regulates urea cycle. (A, B, C) MHCC97H cells were treated with or without 100 μM taurine for four days. Immunoblotting analysis was performed using the indicated antibodies (A). mRNA levels of *GLS1* were analyzed (B). glutamate/glutamine ratio (Glu/Gln) was measured (C). (D, E) MHCC97H cells were treated with or without 1 μM CB839 for 24 h. Immunoblotting analysis was performed using the indicated antibodies (D). mRNA levels of *ASL* were analyzed (E). Data are presented as mean ± SD, n = 3 independent repeats. Unpaired, two-tailed t test. NS, not significant.
