# Supplementary material for: Taurine is a natural suppressor of urea cycle via targeting ASL
Source: Cell Death Discov. 2026 Feb 18;12:99. doi: 10.1038/s41420-026-02959-6 (PMC12921272; doi:10.1038/s41420-026-02959-6)
Supplement: Supplementary file 2 — Uncropped western blots [file 41420_2026_2959_MOESM2_ESM.pdf]

Fig. 1D

Taurine (d)	0	1	2	4
	0.99092	0.73109	0.58448	0.27594
	1.10452	0.71371	0.51615	0.28299
	0.91366	0.68598	0.46788	0.29795

Fig. 1F

sgCtrl	sgPTER
0.977164619	1.70098
0.981733029	1.92197
1.042410707	1.79945

Fig. 3C

Taurine (d)	0	2	4
	0.91053	0.70804	0.42875
	0.9945	0.69297	0.42122
	1.10434	0.69772	0.32726

Fig. 3F

		Vehicle			Taurine	
sgCtrl	0.9877	1.02563	0.98716	0.47833	0.48952	0.50398
sgFOS	0.49078	0.42155	0.39443	0.44309	0.31804	0.36663

Fig. 3G

		IgG			Vehicle			Taurine	
sgCtrl	0.03852	0.03975	0.03524	0.42777	0.45574	0.45576	0.12439	0.14214	0.14119
sgFOS	0.03958	0.03096	0.02547	0.11705	0.12362	0.13202	0.15034	0.11285	0.11107

Fig. S1D

Taurine (d)	0	1	2	4
	0.95901	0.89474	0.74868	0.44435
	1.01766	0.92997	0.8345	0.52288
	1.02464	0.90471	0.70491	0.5366

Fig. S1F

sgCtrl	sgPTER
1.029147342	1.71117
0.965979622	1.71842
1.005899237	1.52193

Fig. S3B

Taurine (d)	0	2	4
	0.92468	0.67054	0.44413
	1.04265	0.60706	0.41187
	1.03721	0.59327	0.39891

Fig. S3D

		Vehicle			Taurine	
sgCtrl	1.01926	0.92642	1.05903	0.52692	0.63047	0.59793
sgFOS	0.50651	0.60514	0.60015	0.50694	0.55702	0.55158

Fig. S3E

	IgG			Vehicle			Taurine		
sgCtrl	0.02676	0.02885	0.02868	0.35598	0.33975	0.36384	0.1116	0.10851	0.11172
sgFOS	0.02888	0.0283	0.03152	0.11097	0.11825	0.09587	0.09717	0.11941	0.1266

Fig. S5B

Vehicle	Taurine
0.974904856	1.05458
0.974904856	0.96371
1.052144848	0.93735

Fig. S5E

Vehicle	CB839
1.049716684	1.06437
0.965936329	0.97942
0.986232704	1.08673

Fig. 1

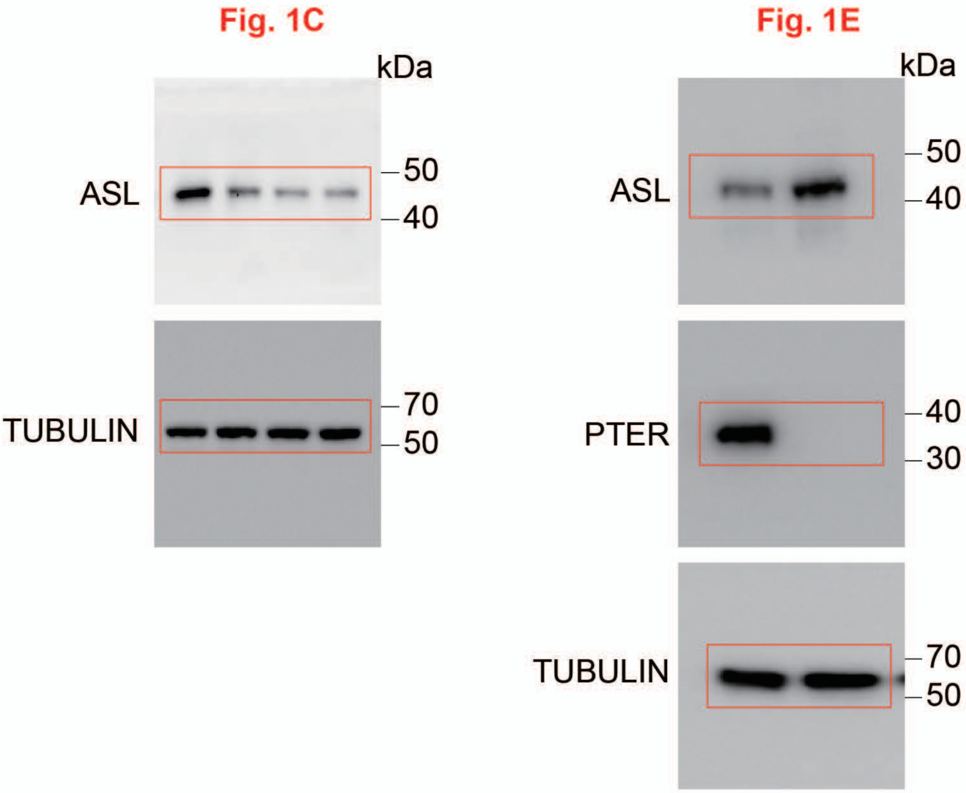
Fig. 1C
kDa
ASL
50
40
TUBULIN
70
50
Fig. 1E
kDa
ASL
50
40
PTER
40
30
TUBULIN
70
50

Fig. 2

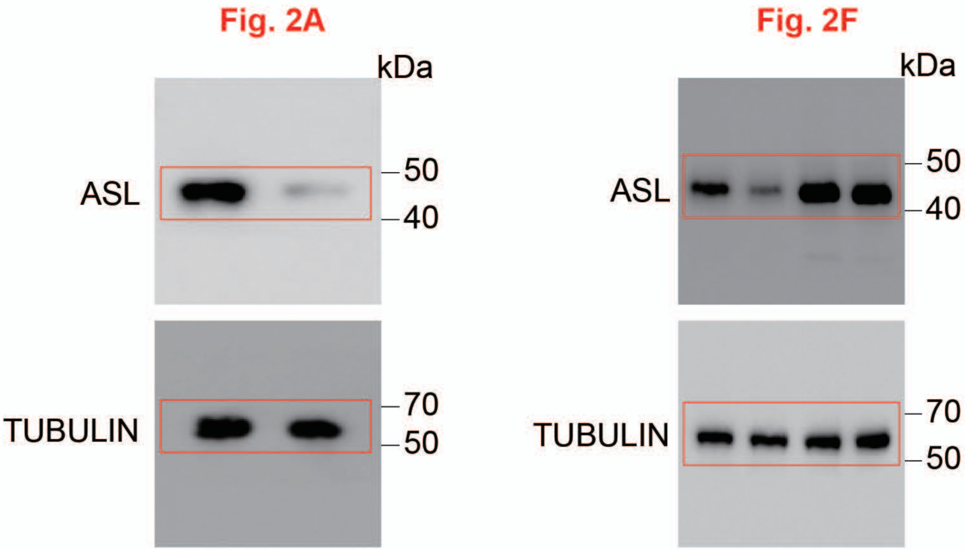
Fig. 2A
kDa
ASL
50
40
TUBULIN
70
50
Fig. 2F
kDa
ASL
50
40
TUBULIN
70
50

Fig. 3

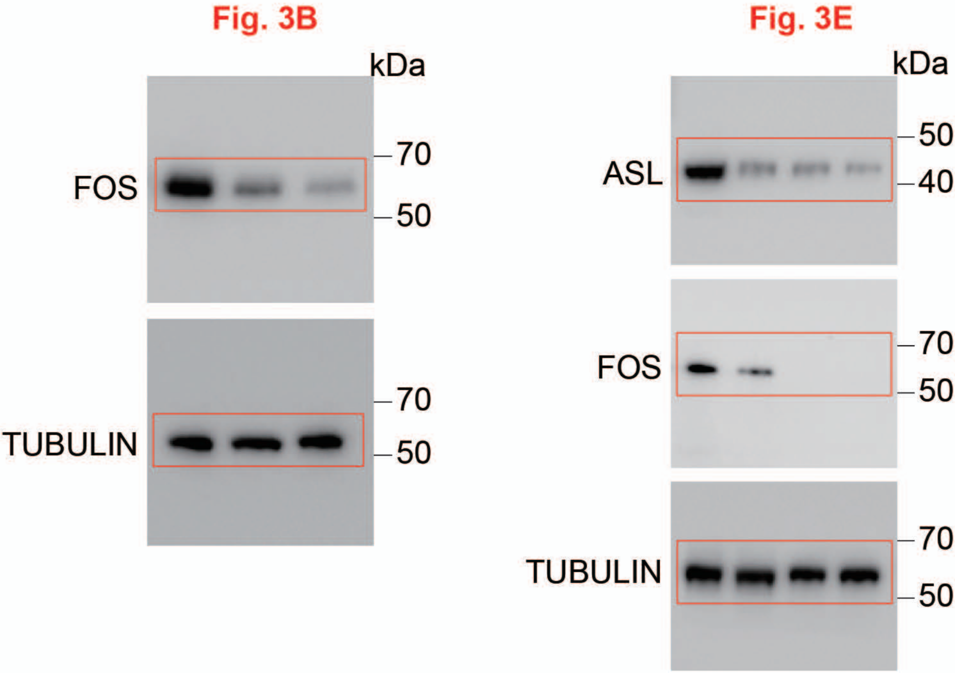
Fig. 3B
kDa
FOS
70
50
TUBULIN
70
50
Fig. 3E
kDa
ASL
50
40
FOS
70
50
TUBULIN
70
50

Fig. 4

Fig. 4A

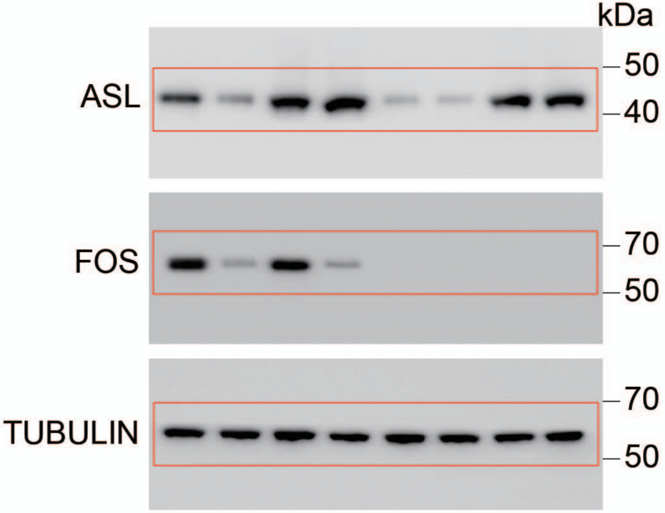

Fig. S1

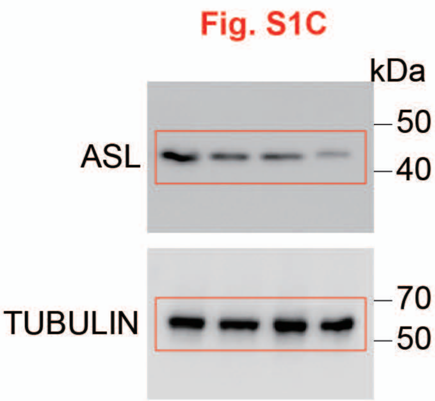

Fig. S1C
kDa
50
ASL
40
70
TUBULIN
50

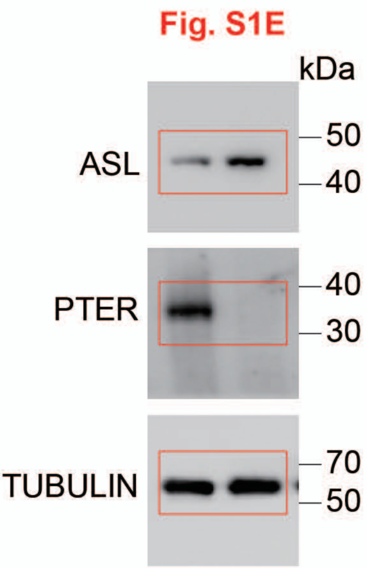

Fig. S1E
kDa
50
ASL
40
40
PTER
30
70
TUBULIN
50

Fig. S2

Fig. S2A

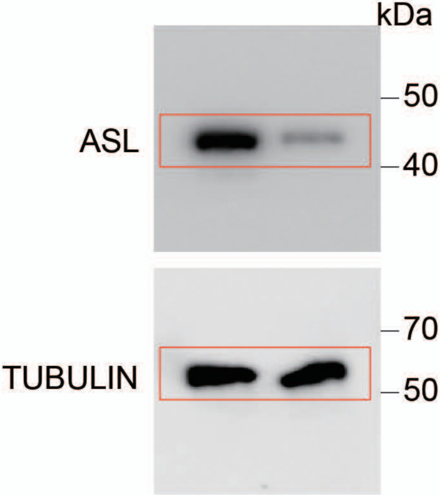

Fig. S2F

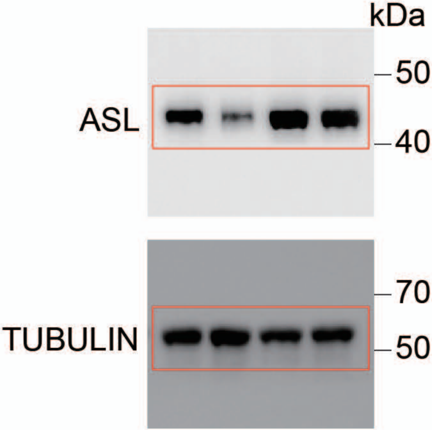

Fig. S3

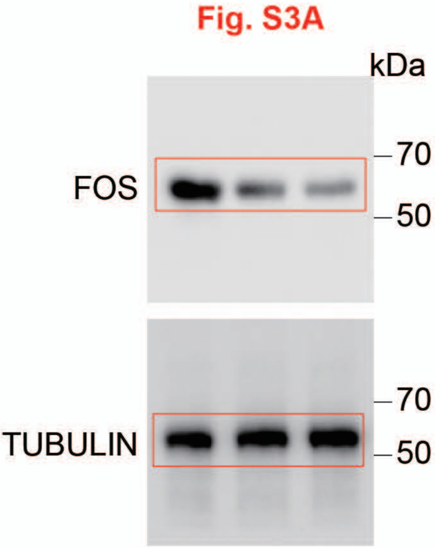
Fig. S3A
kDa
FOS
70
50
TUBULIN
70
50

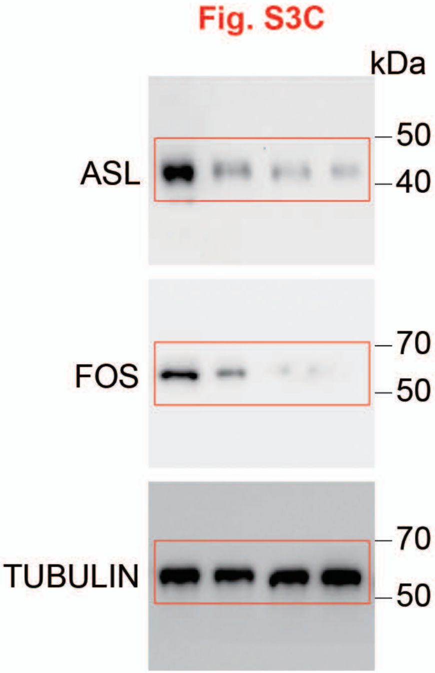
Fig. S3C
kDa
ASL
50
40
FOS
70
50
TUBULIN
70
50

Fig. S4

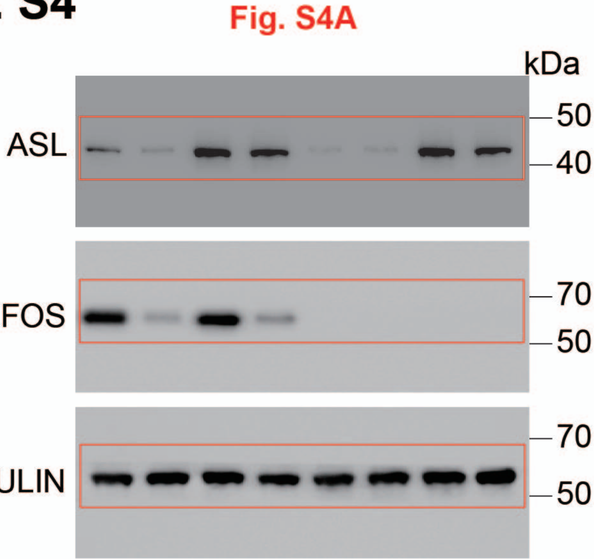

Fig. S5

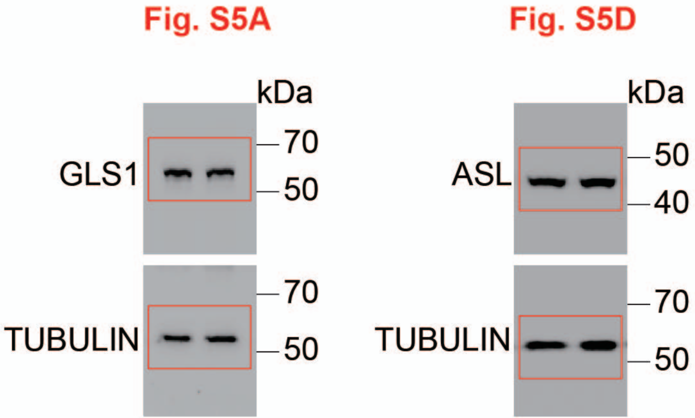
Fig. S5A
kDa
GLS1
70
50
TUBULIN
70
50
Fig. S5D
kDa
ASL
50
40
TUBULIN
70
50
